# The Costimulatory Pathways and T Regulatory Cells in Ischemia-Reperfusion Injury: A Strong Arm in the Inflammatory Response?

**DOI:** 10.3390/ijms19051283

**Published:** 2018-04-25

**Authors:** Laura de Ramon, Jordi Guiteras, Roser Guiteras, Josep M. Cruzado, Josep M. Grinyó, Juan Torras

**Affiliations:** 1 Experimental and Translational Laboratory of Nephrology, Clinic Sciences Department, Universitat de Barcelona, Institut d’investigació biomédica de Bellvitge (IDIBELL), Hospitalet de Llobregat, 08907 Barcelona, Spain; lderamon@idibell.cat (L.d.R.); jguiteras@idibell.cat (J.G.); rguiteras@idibell.cat (R.G.); jmcruzado@bellvitgehospital.cat (J.M.C.); jgrinyo@ub.edu (J.M.G.); 2 Hospital Universitari de Bellvitge, Hospitalet de Llobregat, 08907 Barcelona, Spain

**Keywords:** costimulatory molecules, ischemia, regulatory T cells

## Abstract

Costimulatory molecules have been identified as crucial regulators in the inflammatory response in various immunologic disease models. These molecules are classified into four different families depending on their structure. Here, we will focus on various ischemia studies that use costimulatory molecules as a target to reduce the inherent inflammatory status. Furthermore, we will discuss the relevant role of T regulatory cells in these inflammatory mechanisms and the costimulatory pathways in which they are involved.

## 1. The Importance of Costimulatory Pathways in Ischemia-Reperfusion Injury

Ischemia-reperfusion injury (IRI) is one of the most frequent causes of acute injury that may result in worsening or even loss of organ function [1]. Temporary blocking of the blood flow is followed by a reperfusion period, which aggravates tissue damage by inducing an inflammatory response [2]. Besides the innate inflammatory immune response, there is growing evidence that T cells directly mediate injury in experimental IRI [3]. The CD4^+^ T cells, which work via both interferon-c (IFN-c) and other costimulatory molecules, appear to be important modulators of IRI [4]. Cell stress or tissue damage activates antigen-presenting cells (APCs), leading to upregulation of the costimulatory molecules CD80 or CD40 on their cell surface. These costimulatory molecules then interact with CD28, cytotoxic T-lymphocyte protein 4 (CTLA-4), or CD40L on T cells. 

The understanding of costimulation has emerged from not only positive signals involved in T cell activation but also negative signals inhibiting T cell activation and promoting T cell tolerance [5]. It is suggested that blocking positive or enhancing negative costimulatory pathways may ameliorate organ dysfunction, decrease mononuclear cell infiltration, or expand regulatory T cells (Tregs) in affected ischemic tissue [1].

## 2. Diversity of Costimulatory Molecules

Depending on the structure, costimulatory molecules are classified into four different families: the Ig superfamily, TNF superfamily, integrins or cell adhesion molecules, and TIM molecules.

### 2.1. The Ig Superfamily (IgSF)

This group of cell surface proteins is characterized by the presence of related 70–110 amino acid Ig-like domains and is involved in several cell responses. IgSF proteins have evolved to play key roles in cell adhesion, development, and adaptive immune responses. Many of these IgSF proteins contribute to the immune response through specific cell-to-cell receptor–ligand interactions.

**CD28—B7**: The costimulatory molecule CD28 plays a crucial role in determining T cell sensitivity. CD28 costimulation occurs mainly in the context of inflammation, acting as a mechanism for the activation of T cell response [6]. Blocking the CD28–B7 costimulatory pathway has shown to prevent or delay graft rejection [7,8] by lowering T cell stimulation and, therefore, its clonal expansion [6].

**ICOS—ICOSL**: The CD28 homolog inducible costimulatory molecule (ICOS) is expressed upon activation in CD4^+^ and CD8^+^ T cells. Signaling through ICOS enhances T cell proliferation, survival, and cytokine production.

**PD-1**: Programmed death-1 (PD-1) is a negative costimulatory molecule expressed by T cells, monocytes, dendritic cells, and B cells. PD-1 is indispensable for Treg function as recent studies have revealed that Tregs lacking PD-1 display impaired suppressive activity [9].

**CTLA4**: Cytotoxic T-lymphocyte antigen-4 is an immune molecule expressed on effector and regulatory T cells that can attenuate T cell response by competing for ligands that provide costimulatory signals to T cells via CD28 [10].

### 2.2. The TNF (Tumor Necrosis Factor) Superfamily

Members of the tumor necrosis factor (TNF) superfamily provide costimulatory and/or co-inhibitory signals that are essential for innate and adaptive immunity with an emphasis on T cell response [11]. The extensive sharing of ligands and receptors of this superfamily generates a wide communication network among different T cells and tissues, providing mechanisms for initiating immunity and also resetting homeostasis [11].

Within this large TNF superfamily, we have documented the involvement of CD40, OX40, and CD137 in IRI damage mechanisms [12,13,14]. Remarkably, CD40 was the first member of the TNF receptor family to be recognized. The signaling of CD40 enhances the proliferation of antigen-activated B cells and is driven by the expression of the CD40 ligand in CD4^+^ T helper cells [15]. Moreover, the activation of TNF receptor 2 (TNFR-2) is primarily considered to trigger the pro-survival NF-κB pathway, whereas TNFR-1 activates caspase-dependent pathways [16].

### 2.3. Integrins or Cell Adhesion Molecules

The expression of molecules such as leukocyte function-associated antigen 1 (LFA1) on the cell surface is involved in T cell activation and costimulation [17]. Blockade of LFA1 can prolong pancreatic islet allograft survival [18]. Adhesion molecules are expressed on leukocytes and the endothelial cell surface under the pro-inflammatory milieu [19,20,21].

### 2.4. TIM Molecules

The T-cell immunoglobulin and mucin domain (TIM) family includes TIM-1, TIM-2, TIM-3, and TIM-4 molecules. The TIM family has a wide range of immune functions that include auto and alloimmunity in transplantation, including T cell activation and the clearance of apoptotic cells [22]. For instance, the cross-linking of TIM-1 on CD4^+^ T cells provides a potent costimulatory signal for T cell activation that increases naïve T cell proliferation and interleukin-4 [23]. Moreover, a recent study showed that TIM-1 costimulation prevents allogeneic transplant tolerance by reducing forkhead box p3 (*FoxP3*) expression, thereby preventing the development of Tregs [24].

## 3. Costimulatory Blockade in IRI (Ischemia-Reperfusion Injury)

**Figure 1:**
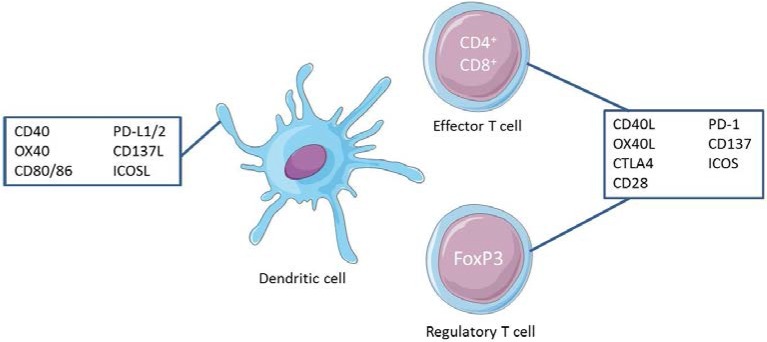
Costimulatory molecules expressed on antigen-presenting cells (APCs) and their ligands on effector and regulatory T cells.

The research of several groups has been focused on targeting costimulatory molecules in ischemia-reperfusion models due to the potential of these molecules to reduce the inflammatory status and at the same time improve organ or graft survival. This overview will focus on ischemia studies with members of both the Ig superfamily and the TNFR family (Figure 1). However, whether the role of costimulation in IRI is comparable in different tissues is a matter that is not clearly defined. We also do not have enough information about the importance of the duration and nature of ischemia, cold or warm, in these costimulatory pathways in IRI.

**CD28–CD80/CD86**: To date, the most well characterized of these costimulatory molecules is CD28, which binds to B7.1 (CD80) and B7.2 (CD86) on the APC surface [25,26]. The binding of CD28 receptors on T cells is a second signal made through T cell receptor (TCR) ligation for naïve T cell activation [27]. Ligation through CD28 increases transcriptional signaling and metabolism and the production of chemokines, cytokines, and survival signals that are crucial for long-term differentiation and the expansion of T cells [28,29,30,31]. Once T cells have been activated, another receptor for CD80/CD86 is expressed, CTLA4. This negative regulatory molecule is structurally homologous to CD28 but has a 10–20-fold higher affinity for B7 proteins than the CD28 molecule. This higher affinity allows CTLA4 to compete with CD28 for its ligand and overturn effector T cell response [32].

The importance of the CD28 costimulatory pathway in the ischemia-reperfusion phenomenon was primarily identified in vivo using the fusion protein CTLA4Ig in an experimental model of renal IRI. This study was the first to confirm early upregulation of the CD28 costimulatory molecule expression in the non-immune acute inflammatory response [33]. The authors used inbred male Lewis rats in a cold ischemia-reperfusion model in which the left kidney was perfused in situ with Wisconsin solution and the right kidney was nephrectomyzed. Animals treated with CTLA4Ig showed reduced mononuclear cell infiltration and activation (ED1^+^ macrophages, CD4^+^ T cells, and MHC class II^+^ cells). Moreover, CTLA4Ig treatment provided complete protection against transient renal ischemia, evidenced by a reduction in serum creatinine compared with control Ig-treated animals [33,34]. The early expression of B7 molecules with no cell infiltration was indicative of endothelial upregulation of the molecule, with B7 blockade inhibiting the adhesion of mononuclear cells in the endothelium and decreasing the tissue infiltration rate.

Later studies were focused on whether B7-1 or B7-2 had a major role on IRI. Using a mutated form of CTLA4Ig binding preferentially to B7-1 did not attenuate acute renal dysfunction as shown by creatinine values [35], indicating a major role of B7-2 in the adverse effects in renal IRI [34]. In contrast, other authors described the deleterious role of B7-1 instead of B7-2, evidenced by the 100 times higher requirement of CTLA4Ig for B7-2 than B7-1 in in vitro studies [36]. In another study involving a warm renal ischemia model, B7-1 was only expressed on endothelial cells of the vasa recta two hours after reperfusion [37], enabling leukocyte adhesion molecule function [38,39,40]. Using a specific B7-1 antibody that neutralizes T-cell adhesion, renal function improved at 24 h after reperfusion, thereby supporting the critical role of T cells in the pathogenesis of IRI as hypothesized by Rabb et al. [37,41].

**ICOS–ICOSL**: ICOS is expressed on the cell surface of activated T cells. Studies in knockout mice for *ICOS* showed that ICOS costimulation is essential for the activation and function of effector T cells [42,43], as well as for the secretion of cytokines, including TNF-α, IL-1, and IL-17 [44,45]. Furthermore, recent studies have demonstrated the connection of ICOS and its ligand in the inflammatory response [45,46]. In an experimental rat model of cerebral ischemia using siRNA against ICOS, the animals showed a significantly lower mortality rate, improved motor coordination, and amelioration of neural tissue damage. In addition, ICOS siRNA decreased the secretion of TNF-α, IL-1, and IL17 from Th1 lymphocytes [47].

Other studies have focused on the progression of atherosclerosis as an event prior to arterial ischemia. These studies have demonstrated that the costimulatory pathway is associated with the proliferation of smooth muscle cells (SMCs) [48,49]. In vitro studies showed a synergistic proliferation in response to IL-1 of SMCs cocultured with activated T cells. The proliferation was decreased using an anti-ICOS antibody, suggesting that SMC proliferation was induced through the ICOS pathway. Furthermore, in vivo studies showed that treatment with the anti-ICOS antibody or ICOS-Ig reduced the development of hyperplasia of the neointima and also in the arteries of *ICOS* knockout mice [50]. Thus, a relationship was found between the ICOS pathway and SMC proliferation in the intima, resulting in the interruption of the blood flow and consequent ischemia by neointimal hyperplasia.

**CD137–CD137L**: These costimulatory molecules have been implicated in multiple stages of inflammation [51,52]. CD137 is expressed in the early inflammatory response on immune cells such as activated T cells, natural killer (NK) or natural killer T cells (NKT), and granulocytes [52,53]. CD137L is mainly expressed on myeloid cells, including professional APCs such as macrophages and dendritic cells [14,54]. CD137L can stimulate CD137 on Th1 helper cells, acting as a regulator of the classical inflammatory pathway secreting various cytokines and chemokines [14].

A mouse model of renal IRI showed how the interaction between CD137 on NK and CD137L on tubular epithelial cells (TECs) boosted the inflammatory response. It is well known that the overproduction of CXC chemokines and their receptors (CXCL1 and CXCL2) by TECS with subsequent neutrophil recruitment results in a cascade of pro-inflammatory events during renal IRI [51,55]. Agonistic monoclonal antibodies (mAbs) for CD137L have been used to prevent several diseases [56,57], suggesting that anti-CD137 mAbs may be used as prophylaxis in ischemic renal failure.

**CD40–CD40L**: CD40 was first identified on B cells but its expression was later localized in several other cells and APCs, such as dendritic cells (DCs), macrophages, and monocytes [58,59,60,61]. CD40L is mainly expressed in CD4 and CD8 activated T cells among other cell types [62,63]. It also has a soluble form, which is mainly expressed on platelets. CD40–CD40L binding has been implicated in T and B cell activation, immunoglobulin switching, germinal center formation, and as inflammatory mediators [64,65,66,67].

In a mouse model of liver ischemia using monoclonal antibodies against CD40L, authors described cytoprotection of the liver. After 90 min of warm ischemia followed by 4 h of reperfusion, sALT levels were significantly increased in non-treated animals. In contrast, sALT levels were reduced in animals in which the CD40L was disrupted by either the use of knockout animals or an antibody against CD40L, thus preventing hepatic ischemic insult [68].

Furthermore, studies with rhesus monkeys undergoing renal transplantation using humanized monoclonal antibodies against CD40L showed long-term animal and graft survival. The animals received a single dose of the mAb every other day, achieving a state in which rejection did not occur even after the drug was withdrawn [69]. However, the anti-CD40L antibodies were later associated with thromboembolism due to platelet activation [70]. These strategies led to the need for other strategies such as the use of CD40 rather than CD40L as a target to prevent allograft survival.

Regarding renal IRI models, several authors have demonstrated the effective role of blocking *CD40*. Rabb et al. showed an overexpression of CD40 mRNA in renal tissue as early as 6 h after ischemia but also after 28 days [71]. Our group has successfully reported CD40 silencing using a small interference RNA (siRNA) in IRI, as well as in other models of disease: acute allograft rejection, atherosclerosis, and autoimmune inflammatory processes [3,12,72].

In rat humoral-like acute vascular kidney rejection, our group administered siRNA locally in a single injection and observed a modification of the rejection pattern on histological evaluation. The siRNA-CD40-treated groups showed a better mean survival time and molecules that are related to the innate response were diminished compared with the non-treated groups [12].

Different doses of siRNA-CD40 have been used in rat kidney ischemia models. Systemic siRNA single injection administration prior to 40 min of warm ischemia showed an amelioration of renal function in treated animals. In addition, the renal inflammatory status was reduced and systemic inflammatory modulation was observed [3]. Additionally, the effect of siRNA-CD40 has been studied in a syngeneic and allogeneic model of kidney transplantation. In the syngeneic model with the addition of IRI, a single dose in siRNA-treated animals showed an amelioration of renal function and a better histologic pattern after 4 days of reperfusion. In the allogeneic model, the animals displayed a partial effect reducing creatinine values. Renal histologic analysis of the surviving rats according to the Banff criteria showed a reduction of inflammation in treated animals [3].

**PD-1–PD-L1**: Programmed death-1 (PD-1) is a CD28 homolog selectively expressed by activated T, B, and myeloid cells [73]. Its ligand PD-L1 is expressed in many cell types including non-immune cells [74], and its interaction delivers negative signals that inhibit T and B cell activation, promoting immune tolerance [75,76]. Haofeng et al. were the first group to use a partial liver warm ischemia model, which by stimulating PD-1 signals demonstrated an amelioration of liver IRI. In this study, it was shown that PD-1–PD-L1 engagement using a recombinant fusion protein [77,78] protected livers from fulminant IRI by a reduction in sALT levels, an amelioration of histological features, a reduction of T cells, inflammatory chemokines, neutrophils and macrophages, and the promotion of local IL-10-dependent cytoprotection [79].

Recently, other authors have also focused on the second ligand of PD-1, PD-L2, the expression of which is limited to APCs [80,81]. In this study, these authors sought to determine the role of both PD-1 ligands in the natural course of kidney IRI and in Treg-mediated protection against IRI [82].

## 4. Role of Tregs in IRI

Different subsets of T cells play diverse key roles in the whole alloreactive response. Specifically, Tregs are described as a functionally distinct T cell subpopulation inducing self-tolerance and homeostasis [83]. As conventional T cells, the Treg population involves T cell receptor and costimulatory molecules for its activation [84]. Binding of identical costimulatory molecules on effector T cells and Tregs can lead to different responses [85]. These were initially described as CD4^+^CD25^HI^, representing the most well-characterized subset of Tregs [86]. Later, the nuclear transcription factor FoxP3 was described as not only a specific marker but also as a modulator of the development and function of these cells [87,88,89,90].

Natural Foxp3^+^ Tregs (nTregs) are mainly produced by the thymus and regulate peripheral self-tolerance. Some of these nTregs are inducible (iTregs) and differentiate from naive T cells in the periphery, being exposed to antigens in the presence of costimulatory molecules [91]. Both nTregs and iTregs have an adaptive immune response suppression function and, consequently, anti-inflammatory properties [92].

Kinsey et al. validated the role of Tregs in acute ischemic renal injury using an antibody against CD25. Mice with a partial depletion of Tregs showed higher neutrophil and macrophage infiltration than non-treated mice after IRI. After transferring Tregs from wild-type (WT) mice into RAG-1 knockout mice (T and B cell deficient strain), inflammatory infiltration decreased in the injured area [93]. The involvement of Tregs during reperfusion has also been demonstrated in a mouse model of intestinal ischemia. In this case, partial depletion of Tregs with an anti-CD25 antibody potentiated intestinal permeability caused by IRI, and different inflammatory markers increased. Moreover, the adoptive transfer of Tregs significantly reduced the intestinal permeability associated with IRI [94]. More recently, in a mouse renal ischemia model, the number of Tregs increased 72 h after reperfusion. In a group of mice in which Tregs were depleted, the number of Tregs at 72 h after reperfusion decreased and, concomitantly, kidney injury and renal function were clearly aggravated. This clearly defines the relationship between the recruitment of Tregs into the kidney and renal function recovery after IRI [95].

Treg recruitment to the injured area is orchestrated by chemokine receptors and adhesion molecules, which are constitutively expressed [96,97]. Furthermore, their immunosuppressive and anti-inflammatory effects are assumed through contact-dependent and soluble mediators [98,99]. Tregs have an inhibitory effect on effector T cells, suppressing their proliferation (by upregulation of CD25 expression), causing cell cycle arrest or apoptosis (by the expression of soluble mediators such as galectin-1, 10 or granzyme A and B), and secreting suppressor cytokines such as IL-10, TGF-β, and IL-35. Moreover, Tregs also have an effect on DCs. They inhibit the maturation of DCs, eluding their interaction with T effector cells [100], through the modulation of DC cell surface expression, and also their secretion of suppressor cytokines. Thus, interplay between Tregs and DCs could suppress the immunostimulatory capacity of DCs and induce the secretion of immunosuppressive cytokines such as IL-10 and TGF-β as well.

## 5. Treg Kinetics Targeting Costimulatory Pathways

**Figure 2:**
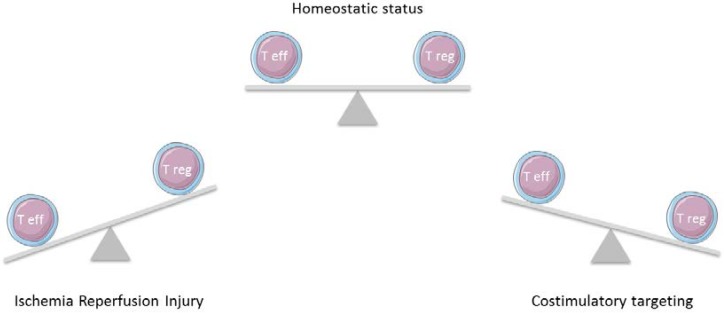
T effector cells have a major role in the inflammatory immune response after ischemic damage. Through modulating the costimulatory pathways, T regulatory cells will exert a strong influence to return homeostasis.

Several studies have consistently shown that modulation of specific costimulation pathways changes Tregs expansion or suppression surpassing their anti-inflammatory effects (Figure 2). However, there are few studies on the topic of the IRI immunoinflammation target, and the data presented here constitute a strong background for future studies on IRI.

### 5.1. Tregs and CD28/CTLA4–B7

In a knockout CD28 mouse model of autoimmune disease with a reduced absolute number of Tregs, the authors observed an increase in autoimmunity response in the animals, suggesting that CD28 is involved in Treg expansion and consequently their function [8]. Furthermore, CD28 blockade (either with anti-B7 mAbs or CTLA4) inhibits Treg homeostatic proliferation and reduces Treg numbers [101,102].

Regarding CTLA4, it has been reported that Tregs need this molecule for immune protection in the kidney [103], and the interaction of Tregs and DCs through CTLA4 downregulates their costimulatory molecules, thereby reducing the inflammatory status [104]. In contrast, a specific deficiency of CTLA-4 in Tregs resulted in the spontaneous development of systemic lymphoproliferation, fatal T cell-mediated autoimmunity, and hyperproduction of immunoglobulin E in mice [102]. Treg-specific CTLA-4 deficiency impaired Treg downregulation of CD80/CD86 expression in DCs in vivo and in vitro [102]. In a model of bilateral IRI in mice, the administration of Treg suppressing agents (either anti-CTLA-4 or anti-CD25 mAb) abolished the renoprotection induced by *N*,*N*-dimethylsphingosine (DMS) [101]. Altogether, these studies validate the critical role of the costimulatory CD28/CTLA4-B7 dyad in Treg homeostasis function.

### 5.2. Tregs and CD40–CD40L

One important effect of this pathway is the enhanced suppressor function acquired by Tregs and the decreasing infiltration of T cells on the inflammatory side, which was seen in a mouse model of skin allograft in which CD40L was blocked [105]. Moreover, in a murine renal transplant model with a single dose of anti-CD40L, the amount of FoxP3 that infiltrated the graft was increased [106].

Some studies have combined the concurrent blockade of various costimulatory pathways. In a full mismatch allogeneic splenocyte transfer mouse model, the authors blocked CTLA4 and CD40L to see how Tregs contribute to immune suppression. The CD40L antibody had an important effect on the CD4 T cell subset whereas CTLA4 had a more relevant effect on the CD8^+^ population, thus suggesting that the combination of both is required for full protection. Interestingly, in this work, different CTLA4 concentrations were used in combination with anti-CD40L. It was seen that low doses of CTLA4 increased Treg expansion whereas high doses of CTLA4 prevented Treg expansion. This could explain some of the controversial results published on CD28 blockade and its effect on Treg populations [107]. 

Concerning the IRI scenario, in a model of right femoral artery ligation, CD28-, B7-1/2-, or CD40-deficient mice showed a significant increase in post-ischemic inflammatory response and vessel growth. This occurred through a profound reduction in the number of Tregs and a concomitant enhanced accumulation of T effector cells and macrophages in the ischemic leg [108]. The infusion of exogenous Tregs or endogenous activation of Tregs abrogated the CD28-deficient splenocyte-induced activation of the inflammatory response and neovascularization [108].

### 5.3. Tregs and OX40–OX40L

The OX40 costimulatory molecule has a dual opposite effect depending on the cell subset. It has a positive function on T effector cells and a suppressor function on Tregs, in both in vitro and in vivo studies [13].

The OX40 costimulatory pathway does not affect Treg viability per se but does affect *FoxP3* gene expression, decreasing the suppressive capacity and also reducing IL-10 secretion [13,109]. Blockade of OX40 may promote immune tolerance by both inhibiting T effector cells and enhancing Treg activity. This costimulatory pathway blockade of OX40 is promising as an important target to avoid graft loss as well as IRI.

### 5.4. Tregs and PD-1–PD-L1⁄PD-L2

PD-1 is expressed in Tregs as a costimulatory molecule, which can bind with its PD-L1 and PD-L2 ligands to inhibit TCR signaling in T effector cells and their cytokine production [80,81]. While different T cells (including Tregs) express PD-L1, PD-L2 is only expressed by APCs. Previous studies have shown that PD-1 pathway blockade leads to an increase in the immune response in both in vivo and in vitro experiments, by a mechanism partially related to the abrogation of the immunosuppressive function of Tregs [110,111,112].

A recent study using mAbs to block PD-L1 and/or PD-L2 in mice showed an increase in kidney IRI damage compared with WT mice. Furthermore, the simultaneous blockade of both signals accentuated the damage when compared with the blockade of either ligand alone. The inefficacy of exogenous Treg transfer in mice in which either PD-1 ligand had been abolished also reveals the essential role of this pathway [9]. Therefore, a complete PD-1 upregulation pathway might be a good strategy to protect against IRI.

### 5.5. Tregs and TIM-1 and TIM-4

It has been shown that the TIM family affects Treg populations, reducing *FoxP3* expression. Upregulation of the TIM-1 pathway using mAbs decreased the Treg suppression function in vitro, concomitantly with an increase in Th-17 differentiation with consequent IL-17 secretion [24]. More recently, a study using mAbs anti-TIM-4 described a significant increase in Treg immunosuppressive function versus a reduction in CD4 T effector cells [113]. The blockade of these pathways does not contribute to increasing the total amount of Tregs but does seem to affect their potency [114].

## 6. Summary and Future Directions

Several experimental studies have suggested that anti-oxidative and anti-inflammatory strategies might be effective in promoting organ protection in the acute phase of ischemia in diverse organs. The failure of clinical trials in ischemia inhibiting the inflammatory response might be in part explained by the indiscriminate nature of these interventions that might have inhibited the activity of parenchymal cells leading to injury exacerbation. Understanding the complex regulatory molecular and cellular mechanisms that trigger an inflammatory response in IRI is crucial for the development of novel fine-tuning immunomodulating strategies that aim essentially to restore the reparative function of parenchymal cells so that they can achieve their main task in protecting the organ. 

Identifying such mechanisms is the only hope to developing fine-tuning immunomodulatory interventions that avoid the detrimental effects of total immunosuppression, which can deactivate all intervening cell types. Changes in Treg numbers or their potency can be derived not only through the previously described pathways but also from signals that remain unknown. In this line, recent literature has shown that costimulatory pathways are highly relevant ways to induce Treg expansion. In the next few years, we will hopefully know more about all these costimulatory pathways, as well as their implications for Treg expansion as a means to improve their function in reducing IRI. The present review has updated all the information available to date in this field.

Treg suppressive function is an emergent approach that should be investigated in new studies. This function can be used for either IRI preconditioning or strategies to reverse IRI damage after it has occurred. Successful targeting in clinical trials of these costimulatory pathways with no collateral damage or effects on other vital processes constitutes the best way to investigate these pathways. These findings will help to design effective ways to modulate the immune response by targeting the suppressive functions of Tregs.
